# Pathological Parasocial Attachment Associated With Suicidal Behavior in a Patient With Schizoaffective Disorder and Borderline Personality Disorder: A Case Report

**DOI:** 10.7759/cureus.111997

**Published:** 2026-07-03

**Authors:** Pranvi Sharma, Valery Bastien, Shahzad Chida, Allison Barrale

**Affiliations:** 1 Psychiatry, American University of Antigua College of Medicine, Osbourn, ATG; 2 Psychiatry and Behavioral Sciences, Interfaith Medical Center, Brooklyn, USA

**Keywords:** borderline personality disorder (bpd), erotomanic delusion, parasocial attachment, psychiatry, schizoaffective depressive disorder, suicide risk

## Abstract

Parasocial attachments are one-sided emotional relationships with public figures that are generally benign and may provide emotional comfort, social connection, and identity development. In vulnerable individuals, however, these attachments may become maladaptive when associated with significant emotional distress, functional impairment, or suicidal behavior. Limited literature exists regarding the role of parasocial attachments in individuals with borderline personality pathology and severe mental illness. We present the case of a 30-year-old woman with schizoaffective disorder, depressive type, and borderline personality disorder who developed a longstanding parasocial attachment to a celebrity figure. For more than 10 years, she maintained an intense emotional investment in the celebrity despite having no personal contact. She acknowledged that the celebrity was unaware of her existence and did not reciprocate her feelings. Nevertheless, she experienced profound distress related to the unattainability of the relationship in the context of chronic feelings of emptiness and fears of abandonment. She presented following a suicide attempt involving the ingestion of apixaban and metoprolol, stating that she no longer wished to live because she could not be with the celebrity. Despite a history of auditory and visual hallucinations involving the celebrity, she denied active hallucinations at presentation. She demonstrated preserved reality testing, recognizing that the relationship was one-sided and unattainable. This case highlights how parasocial attachments may become clinically significant in the setting of underlying psychiatric vulnerabilities. Not all parasocial attachments are clinically significant. However, when they are accompanied by emotional distress, functional impairment, or suicidal ideation, clinicians may benefit from exploring their role in a patient’s presentation. Assessing reality testing can help distinguish maladaptive parasocial attachment from erotomanic or other delusional beliefs. Further research is needed to clarify the relationship between parasocial attachments, borderline personality disorder, and suicidal behavior.

## Introduction

Parasocial attachment is defined as a unidirectional and non-reciprocal emotional connection with public figure(s), such as celebrities, artists, fictional characters, and other famous personas [1]. Often, the individual knows of the public figure, but the public figure remains unaware of the individual. The individual develops a deep bond of intimacy and familiarity with the idealized persona without any reciprocation [2,3].

Parasocial attachments have both positive and negative effects. Benefits include promoting healthy attitudes, reducing loneliness, and identity development [4]. Some studies have suggested that parasocial attachments are associated with reduced feelings of hopelessness and may play a role in self-identity formation [5,6]. However, a false sense of intimacy can also paradoxically exacerbate social isolation and increase depressive symptoms in adults [6]. While parasocial attachments are typically benign, they exist on a spectrum of intensity. In certain individuals, these attachments may become maladaptive, as admiration evolves into emotional dependence that compensates for low self-esteem. This may contribute to significant emotional distress, functional impairment, or increased suicidal behavior [4,6].

Borderline personality disorder is characterized by pervasive disturbances in interpersonal relationships, self-identity, mood reactivity, chronic feelings of emptiness, impulsive behavior, fear of abandonment, self-harm, and suicidality [7,8]. These features may increase vulnerability to intense parasocial attachments, as they can provide a sense of emotional intimacy without the perceived risk of abandonment or rejection associated with reciprocal relationships [2,7,9].

Studies have suggested that parasocial attachments can help fulfill emotional needs, particularly in individuals with lower self-esteem [2]. Attachment-based theories propose that parasocial relationships may serve as compensatory sources of connection when interpersonal attachment needs are unmet. Individuals with insecure attachment styles, greater belongingness needs, and lower self-esteem may therefore be more likely to develop intense parasocial attachments as a means of obtaining perceived emotional closeness [10,11].

Although parasocial relationships have been studied in relation to attachment processes and emotional regulation, limited research has examined their clinical significance in individuals with borderline personality disorder. In particular, the relationship between parasocial attachment, borderline personality disorder, suicidality, and severe psychiatric comorbidity remains poorly understood. We present a patient with schizoaffective disorder, depressive type, and borderline personality disorder who developed a clinically significant parasocial attachment to a celebrity. She subsequently exhibited suicidal behavior related to the perceived unattainability of that relationship.

## Case presentation

The patient is a 30-year-old African American woman with a psychiatric history of schizoaffective disorder, depressive type, and a medical history significant for heart failure with reduced ejection fraction (22%) status post-single-chamber automatic implantable cardioverter-defibrillator placement, hypertension, hyperthyroidism, hyperlipidemia, prior stroke, and deep vein thrombosis on apixaban. She was brought in to the emergency department by Emergency Medical Services after ingesting approximately 42-60 tablets of apixaban 5 mg and 30 tablets of metoprolol succinate 25 mg in an attempt to end her life, stating, “If I can’t be with him, I don’t want to live anymore.”

Her psychiatric presentation was notable for a longstanding romantic fixation involving a well-known celebrity that had persisted for over 10 years. She described an intense, one-sided emotional bond and a belief that they were destined to be together despite having no personal contact with the celebrity. However, she understood that the celebrity did not know her personally and did not reciprocate these feelings. She denied any beliefs involving secret communication, coded messages, or special signalling from the celebrity.

The patient reported a history of auditory and visual hallucinations involving the celebrity. These perceptual disturbances had occurred intermittently over several years and were not exclusively associated with acute mood episodes. However, she denied active auditory or visual hallucinations at the time of presentation. She demonstrated mood lability but denied a history consistent with manic or hypomanic episodes. Depressive symptoms included hopelessness, worthlessness, anhedonia, impaired concentration, and recurrent suicidal ideation. Her history was also notable for childhood sexual abuse, confirmed by collateral information, as well as occasional alcohol and cannabis use.

On admission, mental status examination revealed a cooperative patient with depressed mood, constricted affect, a linear but ruminative thought process, and perseverative romantic thoughts involving the celebrity. Insight was fair, as the patient recognized the pattern as “stuck thinking” and a lack of a fixed belief in mutual love. However, she reported poor control over these thoughts. She endorsed recurrent suicidal ideation with intent. Impulse control was fair on evaluation, but historically poor.

During hospitalization, the patient was diagnosed with borderline personality disorder. She described chronic feelings of emptiness, fear of abandonment, and significant mood reactivity, reporting periods of feeling hopeful and optimistic, followed by rapid shifts to depression and hopelessness. She demonstrated a pattern of intense interpersonal attachments and exhibited splitting behaviors toward members of the treatment team. Following a therapeutic interaction with a medical student, she reported that the conversation had changed her outlook on life and referred to the student as an “angel,” stating that she wished to remain alive because of the support she had received. Within days, however, after being informed that staff could not facilitate communication with the celebrity, she expressed feelings of betrayal, stated that the treatment team wanted her to die, and threatened to stop taking her medications, eating, and drinking. These behaviors were accompanied by recurrent passive suicidal ideation, self-harm behaviors, and a history of a serious suicide attempt.

Additional psychosocial stressors emerged during hospitalization. The patient reported limited social support, stating that her family resided out of state and that she had relocated to New York alone with minimal contact with her family. She was also experiencing housing instability and was residing in a shelter at the time of admission. During individual psychotherapy sessions, she repeatedly stated that she did not necessarily want to die but felt that life lacked meaning if she could not be with the celebrity, stating, “It’s not that I don’t want to be alive, but if I can’t be with him, then I don’t see a reason.” The patient repeatedly described the celebrity relationship as a primary source of meaning, hope, and emotional connection.

A recurring theme throughout treatment involved periods of intense emotional distress, which she referred to as “moments.” During these episodes, she frequently isolated herself, remained in bed for prolonged periods, and refused food, medications, and participation in activities. Although she was often unable to identify specific triggers, she gradually developed greater awareness of these emotional states through psychotherapy. Interpersonal relationships were another prominent theme. The patient described a history of unstable relationships with family members and demonstrated a tendency to prioritize romanticized attachments over her own physical and emotional well-being. Throughout her admission, the patient participated in weekly individual psychotherapy sessions. Treatment incorporated trauma-informed mindfulness, resource-oriented music therapy, and internal family systems-informed interventions. Sessions focused on helping her recognize and manage periods of intense emotional distress. Therapy also emphasized improving self-esteem and identifying coping strategies and sources of support outside of the celebrity-focused attachment. Bilateral beat stimulation was used during episodes she described as “moments” to promote body awareness and emotion regulation. By discharge, she showed greater insight into her emotional experiences and interpersonal patterns. She was also able to distinguish between a healthy interest in the celebrity and an emotionally dependent attachment. She identified future-oriented goals related to employment and creative activities and reported a renewed sense of purpose.

To further evaluate personality pathology, the patient was interviewed using a structured clinical format exploring diagnostic features of borderline personality disorder. The McLean Screening Instrument for Borderline Personality Disorder was administered approximately one month into her inpatient stay, and re-administered one month later. On both occasions, she endorsed eight of ten items, placing her at approximately the 89th percentile relative to a non-clinical comparison sample. Responses were consistent with seven of the nine Diagnostic and Statistical Manual of Mental Disorders, 5th Edition (DSM-5) diagnostic criteria for borderline personality disorder, including unstable interpersonal relationships, recurrent suicidal and self-injurious behavior, impulsivity, inappropriate anger, transient paranoid ideation, dissociative symptoms, chronic feelings of emptiness, and identity disturbance. Collectively, the patient’s clinical presentation, observed interpersonal dynamics, and screening results supported the diagnosis of borderline personality disorder [7].

## Discussion

This case involves a patient with schizoaffective disorder, depressive type, and borderline personality disorder who developed a clinically significant parasocial attachment to a celebrity. She subsequently presented with suicidal behavior. Parasocial attachments exist on a continuum and are typically benign, but this case highlights how emotional dependence may become clinically significant in individuals with underlying psychiatric illness [4,6]. In this case, reality testing remained intact, and the attachment was non-delusional, as the patient recognized that the celebrity neither reciprocated her feelings nor was aware of her existence. This differentiates the presentation from delusional disorder, erotomanic subtype, in which individuals maintain a fixed false belief that another person is in love with them despite contradictory evidence [12]. The attachment was conceptualized as a maladaptive parasocial relationship rather than an erotomanic delusion.

The patient exhibited characteristics of borderline personality disorder, including splitting and polarized thinking, impulsivity, an inner sense of emptiness, fear of abandonment, chaotic romantic relationships, and suicidality [7-9]. Suicidal behavior is a well-known feature of borderline personality disorder and contributes substantially to its morbidity and mortality [13]. In the context of borderline personality disorder, parasocial attachments can provide a sense of closeness while reducing the perceived risk of abandonment inherent in reciprocal interpersonal relationships [2]. In this patient, this dynamic may have contributed to the emotional significance of the attachment alongside other psychiatric factors, including psychotic symptoms [9,12]. The clinical presentation suggests that the attachment may have functioned as an emotionally meaningful but ultimately unattainable relationship, reflecting broader patterns of unstable attachment, fear of abandonment, and interpersonal dysregulation that were evident during her hospitalization. The patient’s history of auditory and visual hallucinations involving the celebrity adds another layer of complexity to the case. These experiences may have contributed to the emotional significance of the attachment. However, they do not appear sufficient to explain the attachment on their own. The attachment remained a central feature of the patient’s presentation even when hallucinations were not present. She also consistently recognized that the celebrity did not know her personally or reciprocate her feelings. This suggests that psychotic symptoms may have influenced the attachment, but were unlikely to fully account for its intensity or its association with suicidal behavior.

The patient’s presentation was difficult to attribute solely to schizoaffective disorder. Although she reported a history of hallucinations involving the celebrity, she maintained reality testing and did not believe that the celebrity knew her personally or was in love with her. Many aspects of the attachment reflected themes that emerged repeatedly during hospitalization, including fear of abandonment, chronic emptiness, unstable relationships, and suicidality following perceived rejection. These observations suggest that both schizoaffective disorder and borderline personality disorder may have contributed to the development and persistence of the attachment.

Psychotherapy sessions provided additional insight into the psychological function of the parasocial attachment. The patient consistently described the celebrity relationship as a primary source of meaning and hope, stating that her desire to live was closely tied to the possibility of being with the celebrity. During psychotherapy, discussions repeatedly returned to the relationship when she described what gave her life purpose and expressed that life felt meaningless if she could not be with him. These observations suggest that the attachment served an important psychological function beyond simple admiration. In the setting of limited social support, housing instability, childhood sexual abuse, and chronic feelings of emptiness, the parasocial attachment may have functioned as a source of emotional support. The patient’s history of childhood sexual abuse by a family member may also have been relevant to understanding her presentation, particularly given the established association between early interpersonal trauma and the development of borderline personality disorder. During hospitalization, she demonstrated a strong reliance on attachment figures for emotional stability and struggled with perceived rejection and separation. These patterns were evident in both her interactions with the treatment team and her emotional investment in the celebrity relationship. Although the specific role of early trauma in the development of the parasocial attachment cannot be determined, it may have contributed to maladaptive attachment patterns, increasing the emotional significance of the relationship. This interpretation is consistent with theories suggesting that parasocial attachments may fulfill unmet interpersonal and emotional needs, particularly among individuals with insecure attachment patterns and low self-esteem [10,11].

The literature examining parasocial attachments in individuals with borderline personality disorder remains limited. This case highlights a potential relationship between borderline personality pathology and the development of clinically significant parasocial attachments. The patient’s suicidal behavior illustrates that even non-delusional parasocial attachments can have serious clinical consequences when combined with underlying psychiatric vulnerabilities. Despite intact reality testing, the patient developed an intense emotional dependence on an unattainable celebrity and ultimately attempted suicide. This case suggests that parasocial attachments may function as maladaptive substitutes for interpersonal relationships in some individuals with borderline personality disorder.

An additional implication of this case concerns the nosological status of maladaptive parasocial attachments. While parasocial attachments are typically regarded as normative and often provide psychological benefits, this case demonstrates that such attachments can, in certain individuals, be associated with significant emotional distress, functional impairment, and even suicidal behavior [14]. Currently, the DSM-5-TR does not specifically address pathological forms of parasocial attachment [7]. Further research is required to clarify whether maladaptive parasocial attachment represents a distinct clinical phenomenon, a manifestation of attachment-related pathology, or a transdiagnostic feature observed across psychiatric disorders. Improved understanding of pathological parasocial attachments, including their prevalence, phenomenology, and clinical consequences, may help guide future assessment and management strategies.

Clinically, this case underscores the importance of assessing parasocial relationships as potential sources of emotional dysregulation or suicidal risk, particularly in patients with borderline personality disorder and other severe psychiatric disorders. Interventions may benefit from incorporating strategies to manage intense attachment needs, such as dialectical behavior therapy skills, cognitive reframing, and psychoeducation about one-sided relationships [9].

This case report has several limitations, including limited generalizability to individuals with borderline personality disorder, schizoaffective disorder, or parasocial attachments, as this report describes a single patient. Additionally, a history of auditory or visual hallucinations involving the celebrity may complicate the distinction between parasocial phenomena and psychotic symptoms. Although the patient met the criteria for borderline personality disorder during hospitalization, the diagnosis was established during a single admission, and longitudinal follow-up data were unavailable. Further research is needed to better characterize the relationship between parasocial attachments, borderline personality disorder, and suicidal behavior.

## Conclusions

This case illustrates a clinically significant parasocial attachment that occurred in the context of a suicide attempt, schizoaffective disorder, and borderline personality disorder. Over time, the attachment became a central source of meaning, hope, and emotional connection for the patient. This case highlights how parasocial relationships may acquire substantial clinical significance in vulnerable individuals. A notable feature of this case was the patient’s intact reality testing. Despite the intensity of the attachment, she recognized that the celebrity neither knew her personally nor reciprocated her feelings, distinguishing the attachment from delusional disorder, erotomanic subtype, and other psychosis-related phenomena. Instead, the attachment appeared to develop within a broader clinical context that included both schizoaffective disorder and borderline personality disorder, as well as longstanding difficulties with attachment, emotional regulation, and interpersonal relationships. From a clinical perspective, this case highlights the importance of exploring not only the presence of parasocial attachments but also the role they play in a patient’s emotional life. Particular attention should be paid to whether the attachment is associated with emotional distress, functional impairment, suicidal ideation, or excessive emotional dependence. Assessing reality testing may also help distinguish maladaptive parasocial attachment from erotomanic or other delusional beliefs. This case also raises important questions about the relationship between parasocial attachments, personality pathology, and suicide risk. Future studies examining parasocial attachments in individuals with borderline personality disorder, psychotic disorders, and other severe mental illnesses are needed. Such work may help improve understanding of the factors that distinguish adaptive attachments from those associated with emotional distress, functional impairment, and suicide risk.
